# Elizabethan Decorations at the Gravesend Hospital

**Published:** 1913-01-04

**Authors:** 


### ELIZABETHAN DECORATIONS AT THE GRAVESEND HOSPITAL.

On Christmas morning, owing to the kindness of friends, each patient in the Gravesend Hospital received a present —a warm garment or some other useful article—and after dinner, which consisted of the usual Christmas fare, the patients received their friends. The tea was generously provided by the Ladies' Association. At half-past three the distribution from the tree in the children's ward was carried out by Mr. H. T. Lukyn-Williams, the house surgeon. The decorations reflected much credit on the matron and nursing staff. The first ward on the right on entering, the "William Tingey" Ward, was full of patients, so that every available bed for male patients was in use on Christmas Day. Sister Tingey and her nurses, together with the patients who had decorated the ward, formed by the entrance a canopy of almond blossom, and the scheme was carried out in festoons suspended from the ceiling, whilst pink shades adorned with almond blossom covered the electric lights.

The sister of the "Princess Alice" Ward, with the help of her nurses and patients, imagined and carried out a rustic scene. An old archway covered with jasmine, from the centre of which hung an old-time lantern, surrounded by holly and mistletoe, stood just by the entrance. Jasmine covered the bed-ropes and window-sills, and the portrait of H.R.H. Princess Alexander of Teck, which faces the entrance to the ward, was encircled by the same flower. A huge palm stood on the doctors' table supported by narcissi. The patients' tea-table was very prettily decorated with birds'-nest jasmine baskets, filled with chocolates and cakes. Ivy and roses greeted the eye in the children's ward, thanks to Sister Theatre and her nurses. Garlands stretched across the ceiling, from which hung strands of ivy intermixed with roses. The arches were decorated in harmony, while a "Teddy Bear" climbed a cork-covered pillar. A huge and heavy-laden Christmas tree stood in the centre of the ward, sparkling with coloured electric lights. The Russell Wards and Out-patients' Hall were very tastefully decorated by Sister Surgery and her nurse with evergreens and japonica, with butterflies intermingled and Chinese lanterns.

The night sister and the night nurses had transformed the board-room, where tea was served to the visitors, to represent an Elizabethan cottage, with its old diamond-shaped windows and a very effective imitation grandfather's clock. On Boxing Day the patients again had their friends, and on Saturday evening those well enough were moved to the Out-patients' Hall, where they had a capital entertainment kindly arranged by Miss Stephenson.

